# A Retrospective Analysis of the Disruptions in the HIV Continuum of Care During the COVID-19 Pandemic: Lessons From a Clinic-Based Study

**DOI:** 10.7759/cureus.53416

**Published:** 2024-02-01

**Authors:** Toni-Ann J Lewis, Michael E Kaiser, Natalya Goldshteyn, Douglas Sepkowitz, William M Briggs

**Affiliations:** 1 Internal Medicine, New York-Presbyterian Brooklyn Methodist Hospital, New York, USA; 2 Internal Medicine, St. George's University School of Medicine, Brooklyn, USA; 3 Infectious Disease, New York-Presbyterian Brooklyn Methodist Hospital, Brooklyn, USA; 4 Statistics, Weill Cornell Medical College, New York, USA

**Keywords:** covid 19, : telehealth, covid-19 pandemic, covid-19 telemedicine, covid nyc, continuum of care, treatment outcomes of hiv patients, people living with hiv (plwh), hiv continuum, hiv aids

## Abstract

Background: The COVID-19 pandemic profoundly affected healthcare services, including HIV patient care. This study assessed the impact of the pandemic on diverse aspects of care for individuals living with HIV (PLWH).

Methods: Patient data from 2019 to 2021 were collected using the Cascades template, provided by the New York State Department of Health, focusing on viral testing and suppression outcomes. Age, ethnicity, sex, and race were considered variables and analyzed via chi-square analysis, logistic regression model, and F test.

Results: The pandemic significantly reduced viral testing in 2020 due to restrictions and closures, but telemedicine and tele-pharmacy helped maintain care. Age was a crucial factor, predicting higher viral testing and suppression odds for older individuals, but no significant differences were observed between patient gender, race, or ethnicity in obtaining viral testing or achieving suppression.

Conclusions: While limitations existed, this study provides insights into sustaining care during crises, highlighting the importance of innovative healthcare delivery methods and age-sensitive approaches for PLWH.

## Introduction

On March 11, 2020, the World Health Organization (WHO) officially declared the coronavirus disease 2019 (COVID-19) a global pandemic. By December 23, 2022, the pandemic had encompassed over 740,800 reported cases across more than 228 countries and territories. The ensuing circumstances led to many challenges in the healthcare landscape, with numerous healthcare facilities shutting their doors and individuals being advised to remain at home to shield themselves and others from potential exposure.

Of particular concern were the implications for individuals living with HIV (PLWH), who heavily rely on healthcare providers for essential medical treatment and critical testing. Among PLWH, the achievement and sustained maintenance of viral suppression hold paramount importance, as they are associated with improved overall health outcomes, increased life expectancy, and the remarkable benefit of significantly reduced transmission risk [1,2].

As the world grappled with the challenges posed by the COVID-19 pandemic, individuals living with HIV were particularly susceptible to the upheaval in healthcare services and access. Within this context, the present study was designed to evaluate the multifaceted consequences of the pandemic on the diverse aspects of HIV patient care. Specifically, we sought to comprehensively assess the repercussions on access to medical services, testing, and treatment for PLWH of varying demographic groups in the wake of the pandemic's disruptive influence. Furthermore, our study aimed to identify potential strategies and solutions that could effectively mitigate the challenges and disruptions faced by this vulnerable population during these unprecedented times and to illuminate avenues through which the healthcare community can provide steadfast support to PLWH.

## Materials and methods

Patient data for this study were collected using the Cascades analysis template, endorsed by the New York State Department of Health (NYSDOH) and tailored to organizations providing medical care to PLWH in New York State. This template, closely affiliated with the AIDS Institute's HIV Quality of Care Program annual review, facilitated a comprehensive assessment of the care continuum, from initial linkage and engagement in care to the crucial goal of achieving viral suppression. By applying this template to our patient clinic population, we could effectively track and evaluate the impact of the COVID-19 pandemic in 2020 on patients already established within our clinic and receiving antiretroviral therapy (ARV). Specifically, we explored whether these patients received essential viral load testing (VL) or maintained viral load suppression (VS) compared to the preceding years of 2019 and 2021.

Our focus rested on "established clinic patients," defined as those who had undergone at least two visits to our Infectious Disease clinic for HIV management in the years leading up to the COVID-19 outbreak. By narrowing our analysis to this group, we aimed to capture a more comprehensive view of the pandemic's effects on individuals already engaged in our clinic's care.

Our study encompassed a cohort of 1498 patients undergoing ARV within our Infectious Disease clinic between 2019 and 2021. The Cascades template allowed us to stratify patients based on key demographics, including age categories (20 to 60 years or older), gender (e.g., female or male), race (e.g., Black, White, Asian), and ethnicity (e.g., Hispanic or Non-Hispanic). To mitigate potential information bias, "unknown" variables were categorized as missing during the analysis, preventing inconsistencies in the data reports.

Viral load testing status was evaluated using a binary "YES" or "NO" designation obtained through a comprehensive review of individual patient charts. Meanwhile, VS was defined as having a viral load measurement of <200 copies/mL of HIV using the HIV nucleic acid amplification test (NAAT).

We employed rigorous statistical methods to ascertain the potential impact of the COVID-19 pandemic and specific demographic factors on the likelihood of patients undergoing viral testing or achieving suppression. A logistic regression model set at a 95% confidence interval was utilized to assess the overall relationship between the pandemic and these outcomes. Additionally, the F-test was applied to gauge relationships within demographic groups. The chi-square test of independence, with a significance level set at p <0.05, was employed to explore potential associations between the COVID-19 pandemic, demographic characteristics, and the likelihood of viral testing or suppression.

In essence, our study hinged on the robust utilization of the Cascades analysis template, offering a comprehensive framework for evaluating the effects of the pandemic on PLWH care outcomes. Through rigorous statistical analyses, we sought to uncover insights into the influence of the pandemic and demographic factors on viral testing and suppression, contributing to the broader understanding of healthcare delivery during unprecedented circumstances.

## Results

Viral load testing status between ethnicity, sex, race, and year

We thoroughly examined VL status within specific demographic categories in 2019, 2020, and 2021. Patient characteristics, including ethnicity, sex, and race, defined these categories. The results are as follows and are summarized in Table 1.

**Table 1:** Results of chi-square and logistic regression analysis for patient ethnicity, sex, race, and year with the association of viral testing The data have been presented at a 95% confidence interval. P-value < 0.05 indicates statistical significance. The logistic regression "Reference” represents the probability of viral load testing belonging to that class.

Viral load testing by demographics and year								
Chi-square, p < 0.05					Logistic regression, 95% CI			
Variable	No	Yes	Prediction	P-value	OR	Low	High	P-value
Ethnicity, N = 1175								
Hispanic, n = 252	13 (30.2%)	239 (21.1%)	0.938	0.21	Reference			
Non-Hispanic, n = 923	30 (69.8%)	893 (78.9%)	0.969		1.62	0.805	3.09	0.156
Sex, N = 1497								
Female, n = 648	22 (43.1%)	626 (43.3%)	0.968	1	Reference			
Male, n = 849	29 (56.9%)	820 (56.7%)	0.969		0.994	0.56	1.74	0.983
Race, N = 1333								
Asian, n = 13	0 (0%)	13 (1.01%)	0.987	0.8	Reference			
Black, n = 918	28 (68.3%)	890 (68.9%)	0.972		2.03e^-06^	NA	1.66e^+14^	0.984
White, n = 402	13 (31.7%)	389 (30.1%)	0.972		1.91e^-06^	NA	7.02e^+09^	0.984
Year, N = 1498								
2019 and 2021, n = 891	20 (39.2%)	871 (60.2%)	0.974	0.0043	Reference			
2020, n = 607	31 (60.8%)	576 (39.8%)	0.951		0.427	0.237	0.75	0.00351

Ethnicity

With N = 1175, the analysis yielded a chi-square p-value of 0.21. Upon further segmentation into non-Hispanic versus Hispanic groups, the odds ratio (OR) was calculated to be 1.62, with a 95% confidence interval (CI) ranging from 0.805 to 3.09. The associated p-value is 0.156. These results indicate no statistically significant association between ethnicity and the likelihood of patients receiving VL.

Sex

The chi-square p-value for sex was 1.0 with N = 1497. Upon comparing males to females, the OR was computed as 0.994, with a 95% CI spanning from 0.56 to 1.74. The corresponding p-value is 0.983. This indicates no statistically significant association between a patient's sex and their likelihood of undergoing VL.

Race

Upon assessing race at N = 1333, the chi-square p-value obtained was 0.8. The analysis involved comparing black individuals and Asians, resulting in an OR of 2.03e^-06^, but with a notably wide confidence interval [NA, 1.6e^+14^]. The associated p-value is 0.984. Similarly, when comparing white individuals to Asians, the OR was 1.91e^-06^, with a confidence interval of [NA, 7.02e^+09^] and a p-value of 0.984. Overall, the insignificance of the p-values and the wide confidence intervals in each race-based comparison suggest no statistically significant association between a patient's race and their likelihood of receiving VL.

The analyses of patient ethnicity, sex, and race revealed no statistically significant associations with the likelihood of undergoing VL. The obtained p-values and confidence intervals across these categories consistently suggest that these demographic factors do not strongly predict a patient's engagement in VL.

The data reveal a noteworthy disparity by using chi-square analysis to compare VL across 2019 and 2021 against 2020 at N = 1498. More patients did not undergo testing in 2020 (60% in 2020 compared to 39% in 2019 and 2021). This observation is underscored by a p-value of 0.043, indicating statistical significance. Consequently, an association between 2020 and patients' likelihood of undergoing viral testing is discernible.

Further investigation through a logistic regression model corroborates these findings. The OR was calculated to be 0.427, with a 95% CI spanning from 0.237 to 0.75. The accompanying p-value of 0.0035 supports the statistical significance of the association between patients receiving viral testing in 2020. If all other variables are held constant, these results imply that individuals were 57.3% less inclined to undergo viral testing in 2020 than in the preceding years, namely 2019 and 2021.

Viral suppression status between ethnicity, sex, race, and year

When examining the suppression status of individuals within the same demographic categories, we found the following results (Table 2).

**Table 2:** Results of chi-square analysis and logistic regression for patient ethnicity, sex, race, and year with viral suppression The data have been presented at a 95% confidence interval. P-value <0.05 indicates statistical significance. The logistic regression “Reference” represents the probability of viral suppression belonging to that class.

Viral suppression by demographics and year									
Chi-square, p < 0.05						Logistic regression, 95% CI			
Variable	N	No	Yes	Prediction	P-value	OR	Low	High	P-value
Ethnicity, N = 1129									
Hispanic, n= 238		24 (19%)	214 (21.3%)	0.899	0.63	Reference			
Non-Hispanic, n= 891		102 (81%)	789 (78.7%)	0.886		0.868	0.532	1.37	0.553
Sex, N = 1442									
Female, n= 629		79 (47%)	545 (42.8%)	0.872	0.34	Reference			
Male, n= 818		89 (53%)	729 (57.2%)	0.896		1.19	0.859	1.64	0.297
Race, N = 1290									
Asian, n= 13		0 (0%)	13 (1.14%)	0.982	0.31	Reference			
Black, n= 888		111 (72.1%)	777 (68.4%)	0.876		1.22e-06	NA	1,590,000	0.973
White, n= 389		43 (27.9%)	346 (30.5%)	0.882		1.40e-06	NA	1,750,000	0.973
Year, N = 1473									
2019 and 2021, n= 898		99 (58.9%)	769 (60.3%)	0.888	0.79	Reference			
2020, n= 575		69 (41.1%)	506 (39.7%)	0.88		0.944	0.682	1.31	0.73

Ethnicity

The chi-square p-value for ethnicity at N = 1129 was 0.63. When comparing non-Hispanic individuals to Hispanics, the OR was calculated to be 0.868, with a 95% CI ranging from 0.532 to 1.37. The associated p-value is 0.553. These findings indicate no statistically significant association between ethnicity and the likelihood of achieving viral suppression.

Sex

The chi-square p-value for sex was determined to be 0.34 at N = 1442. Upon comparing males to females, the calculated OR was 1.19, with a 95% CI from 0.859 to 1.64. The corresponding p-value is 0.297. These results suggest no statistically significant association between a patient's sex and their likelihood of achieving viral suppression.

Race

At N = 1290, the chi-square p-value for race was 0.31. When comparing black individuals to Asians, the OR was computed as 1.22e^-06^, but with a wide confidence interval [NA, 15.9e^+65^]. The associated p-value is 0.973. Similarly, when comparing white individuals to Asians, the OR was 1.40e^-06^, with a confidence interval of [NA, 17.5e^+6^] and a p-value of 0.973. As with the viral testing analysis, these results indicate no clear evidence of an association between a patient's race and their likelihood of achieving viral suppression.

The comparisons of suppression status within the demographic categories of ethnicity, sex, and race revealed no statistically significant associations. The obtained p-values and confidence intervals across these categories consistently suggest that these demographic factors do not play a substantial role in determining a patient's likelihood of achieving viral suppression.

We examined the relationship between different years (2019, 2020, and 2021) and the status of viral load detection and suppression rates among patients with N = 1473. Our analysis revealed variations in the percentage of patients with detectable viral loads during different years. In 2019 and 2021, 58% of patients had detectable viral loads, whereas this percentage decreased to 41% in 2020. This indicates a potential impact of the COVID-19 pandemic on viral load monitoring and control. Despite the observed differences in detectable viral loads, the association between the year and VS rates was not statistically significant. The calculated p-value for this comparison was 0.79, suggesting that the observed differences in suppression rates between 2019, 2020, and 2021 are likely due to random variability rather than a true association. The OR analysis was used and aimed to determine the likelihood of an individual achieving viral suppression in 2020 compared to the combined years 2019 and 2021. The calculated OR was 0.994, and the 95% confidence interval ranged from 0.682 to 1.31. The associated p-value for this comparison was 0.73, which is not statistically significant. These results indicate no substantial difference in the odds of achieving VS between 2020 and the combined years 2019 and 2021.

While there were fluctuations in the percentage of patients with detectable viral loads during different years, our analysis did not identify a statistically significant association between the year and decreased VS rates. The odds of achieving VS in 2020 were slightly lower than those in the combined years 2019 and 2021. However, these differences were not statistically significant, as indicated by the obtained p-value and confidence interval. Therefore, our data do not provide strong evidence to suggest that the year 2020 had a significant impact on VS rates among the studied patient population.

Viral testing and suppression status for age

We observed significant associations between age, viral testing, and suppression outcomes when incorporating age as a variable in our analysis. The analysis revealed that age significantly predicts the likelihood of individuals undergoing viral testing. The average age of the studied population was approximately 55-56 years old. For every one-year increase in age, the odds of an individual receiving viral testing increased by approximately 2%. This association's calculated OR was 1.02, with a 95% confidence interval ranging from 1.00 to 1.04. The p-value associated with this analysis was 0.0504, indicating a statistically significant relationship between age and the likelihood of receiving viral testing (Table 3). Similarly, age was found to be a significant predictor of viral suppression outcomes. For each additional year of age, the odds of an individual achieving VS increased by 4%. The OR associated with this relationship was 1.02, and the 95% confidence interval ranged from 1.03 to 1.06. The p-value for this association was exceptionally low, at 6.94e^-12^, indicating a robust and highly statistically significant link between age and the odds of achieving viral suppression (Table 4).

**Table 3:** F-test and logistic regression analysis showing a statistically significant relationship between age and the likelihood of receiving viral testing. The data have been presented at N = 1498, mean = 55.1 ± 13.1. P-value < 0.05 indicates statistical significance.

Viral load testing by age, N = 1498									
F-test, p < 0.05						Logistic regression, 95% CI			
Variable	Median	Mean	Standard deviation	Prediction	P-value	OR	Low	High	P-value
Age									
No	52.7	51.4	14.3	0.964	0.0495	1.02	1	1.04	0.0504
Yes	56.9	55.1	13.1	0.964					

**Table 4:** F-test and logistic regression analysis showing age to be a significant predictor of viral suppression. The data have been presented at N = 1498, mean = 56 ± 12.9. P-value < 0.05 indicates statistical significance.

Viral suppression by age, N = 1498									
F-test, p < 0.05						Logistic regression, 95% CI			
Variable	Median	Mean	Standard deviation	Prediction	P-value	OR	Low	High	P-value
Age									
No	50.4	48.4	13.1	0.858	1.47e^-12^	1.04	1.03	1.06	6.94e^-12^
Yes	57.6	56	12.9	0.902					

Our analysis demonstrated that age substantially impacts both viral testing and viral suppression outcomes among the studied patient population. Older individuals were more likely to undergo viral testing, with a 2% increase in testing odds for each additional year. Furthermore, older individuals were also more likely to achieve VS, with a 4% increase in suppression odds for each year of age. These findings underscore the significance of age as a predictor in the continuum of care for people living with HIV, particularly in the context of viral testing and suppression outcomes. 

## Discussion

In conclusion, the results of chi-square analysis and logistic regression for patient ethnicity, sex, race, and year with viral suppression revealed no statistically significant associations, suggesting that these factors do not strongly influence the likelihood of viral suppression among patients nor as effective predictors for the probability of patients achieving viral suppression. There was also no significant association between 2020 and patients obtaining viral suppression, nor any significant association in the likelihood that a patient would have VS in 2020 compared with 2019 and 2021. A similar analysis for patient ethnicity, sex, race, and year with the association of viral testing paralleled results. It showed no statistically significant associations, suggesting that these factors do not strongly influence or serve as effective predictors of the probability of viral testing. There was a significant association between 2020 and the likelihood of patients receiving viral testing; patients were 57.3% less likely to undergo testing in 2020 than in 2019 and 2021. Interestingly, the F-test and logistic regression analysis show a statistically significant relationship between age and the likelihood of patients receiving viral testing and suppression.

During our analysis, a paramount observation emerged: specifically, the pivotal role played by the COVID-19 pandemic in 2020. This year exhibited a statistically significant connection with a reduction in the number of patients receiving viral testing, which can be largely attributed to the intricate web of pandemic-related constraints, including factors like laboratory closures that inevitably led to delayed HIV surveillance [3]. In March 2020, the implementation of comprehensive community containment measures, encompassing social distancing, movement restrictions, and quarantine mandates for suspected or confirmed cases, further underscored the extraordinary circumstances brought about by the pandemic [4,5]. These limitations significantly impeded routine HIV testing and management, creating substantial barriers for patients accessing essential healthcare services, including viral testing.

Our findings align with the broader global landscape, where analogous patterns have been observed. In a study examining the impact of the COVID-19 pandemic on PLWH in Belgium during the initial wave, a notable decrease in new HIV diagnoses and consultations emerged [6]. Likewise, a similar trend was evident in Northern Italy, where the occurrence of new HIV cases declined when compared to the average monthly tally of new diagnoses in 2019 [7]. A Boston Community Health Center serving people living with HIV saw a significant decrease in in-person visits and a subsequent increase in telehealth services in the beginning months of 2020. When telemedicine and in-person visits were combined, the mean number of visits per month by PLWH was higher in 2020 when compared to the same period in 2019 [8]. These observations echo our own and are plausibly attributed to the enforced clinic closures and stringent government-mandated isolation directives.

Notably, our analysis underscores the significant role telemedicine appointments assumed during the tumultuous pandemic. Despite the adversities imposed by COVID-19, telemedicine served as a crucial lifeline, facilitating the continuity of routine follow-up appointments and ensuring the preservation of the status quo for viral testing and suppression. Prior studies suggest that missed medical appointments are independently associated with an increased risk of AIDS-defining illnesses and death [9, 10]. In the face of the widely advocated #stayathome approach, telemedicine emerged as an ingenious solution to bridge the gap between the need for medical assistance and minimal physical contact [11]. Retention in care is essential for the health of PLWH and public health [12], and by embracing telehealth solutions, patients could engage in vital consultations, aiding in maintaining adherence to ARV and consequently promoting better health outcomes [13]. 

Moreover, the significance of telemedicine extended beyond the maintenance of care to encompass the achievement of viral suppression across diverse demographic categories. This serves as a testament to the efficacy of these innovative strategies and the resilience of patients and healthcare providers alike. Notably, the utilization of specialty pharmacy-driven home delivery of antiretroviral drugs (ARVs) emerged as a formidable tool in this context. This practice, which ensured uninterrupted access to essential medications, played an instrumental role in averting potential disruptions in the monitoring of PLWH in terms of viral load and suppression [14]. By harnessing this approach, pharmacists could seamlessly extend their expertise remotely, ensuring medication accessibility and the sustenance of viral suppression [15]. These streamlined delivery methods stood as a lynchpin in maintaining a consistent level of care, underscoring their significance as a potential cornerstone in addressing future healthcare challenges [16]. Exploring long-term telemedicine/tele-pharmacy solutions strengthens the medical infrastructure and may be critical in negating any consequences of unforeseen medical emergencies. 

The age-related aspect of our study surfaced as another significant dimension. The influence of age on HIV care and outcomes was unmistakable, with older individuals, averaging 55-56 years, displaying higher tendencies toward viral testing and suppression. For every year increase in age, the odds of receiving viral testing increased by approximately 2%, and the odds of achieving viral suppression increased by 4%. People more than 50 years of age now account for almost one in six people with a new HIV infection in Canada and the United States [17, 18]. This phenomenon can likely be attributed to the accumulated experience and familiarity with medication regimens that typically accompany advancing age [19]. Although not clinically significant, our findings thus emphasize the necessity of considering age-related factors when formulating tailored interventions for HIV care, especially in times of crisis. Understanding the interplay between age-related variables and adherence is paramount, and healthcare providers must adopt a comprehensive approach that addresses the gamut of personal, psychological, and social factors influencing adherence to ARV.

Acknowledging the limitations inherent in our data collection and analysis process is prudent. Data gathering was not immune to challenges, such as incomplete information due to limited access to viral test results from external private practices or introduction of a new Electronic Medical Records system in December 2021. Additionally, the impact of the pandemic on clinic operations during the period from April 2020 to February 2021 led to data disruptions, impacting the ability to collect comprehensive patient data during this period. 

## Conclusions

In summary, the COVID-19 pandemic prompted the implementation of innovative measures that bolstered ongoing care and sustained viral suppression outcomes for individuals living with HIV. Notably, 2020 exhibited a statistically significant correlation with reduced viral testing, a trend likely influenced by limited healthcare accessibility during the pandemic's restrictions. However, telemedicine and telepharmacy solutions effectively preserved patients' engagement in our practice, ensuring uninterrupted viral suppression and testing, irrespective of gender, race, or ethnicity. Moreover, our analysis highlighted the significant predictive role of age in both viral testing and suppression. This underscores the importance of factoring age-related considerations into HIV care planning. Recognizing these insights, our clinic has embraced a range of measures to enhance compliance and medication adherence. These measures encompass expediting clinic appointments, initiating antiretroviral treatment before viral resistance assessment, and facilitating home deliveries of medication through specialized pharmacies, particularly in unique cases. We have also expanded the availability of social work services to mitigate issues related to clinic access and patient insurance challenges. Additionally, a diversified array of follow-up options, including virtual platforms like Zoom, phone consultations, and in-person visits, are being implemented to accommodate diverse schedules, occupational demands, and mobility constraints. By intensifying counseling and involving social workers, we intend to address the concerns arising from the apprehensions posed by the pandemic.

Despite the disruptive impact of the pandemic, these concerted strategies have empowered healthcare providers to deliver indispensable services to PLWH consistently. Consequently, continuity of care and sustained viral suppression outcomes have been upheld. These findings underscore the imperative of adaptive healthcare delivery methods to effectively navigate unexpected challenges, all while prioritizing patient-centric care in the face of evolving circumstances.
